# Crystal structure of 2-amino-4-methyl­pyridin-1-ium (2*R*,3*R*)-3-carb­oxy-2,3-di­hydroxy­propano­ate monohydrate

**DOI:** 10.1107/S160053681401842X

**Published:** 2014-08-23

**Authors:** J. V. Jovita, S. Sathya, G. Usha, R. Vasanthi, A. Ramanand

**Affiliations:** a PG and Research Department of Physics, Queen Mary’s College, Chennai-4, Tamilnadu, India; b Department of Physics, Loyola College, Chennai-34, Tamilnadu, India

**Keywords:** crystal structure, 2-amino-4-methyl­pyridin-1-ium, tartrate, l-(+)-tartaric acid

## Abstract

The title mol­ecular salt, C_6_H_9_N_2_^+^·C_4_H_5_O_6_^−^·H_2_O, crystallized with two 2-amino-4-methyl­pyridin-1-ium cations, two l-(+)-tartaric acid monoanions [systematic name: (2*R*,3*R*)-3-carb­oxy-2,3-di­hydroxy­propano­ate] and two water mol­ecules in the asymmetric unit. In the crystal, the cations, anions and water mol­ecules are linked *via* a number of O—H⋯O and N—H⋯O hydrogen bonds, and a C—H⋯O hydrogen bond, forming a three-dimensional structure

## Related literature   

For the biological activity of pyridinium derivatives, see: Judge & Bever (2006 ▶); Schwid *et al.* (1997 ▶); Strupp *et al.* (2004 ▶). For the crystal structure of a related mol­ecular salt involving the 2-amino-4-methyl­pyridin-1-ium cation, see: Hemamalini & Fun (2010 ▶).
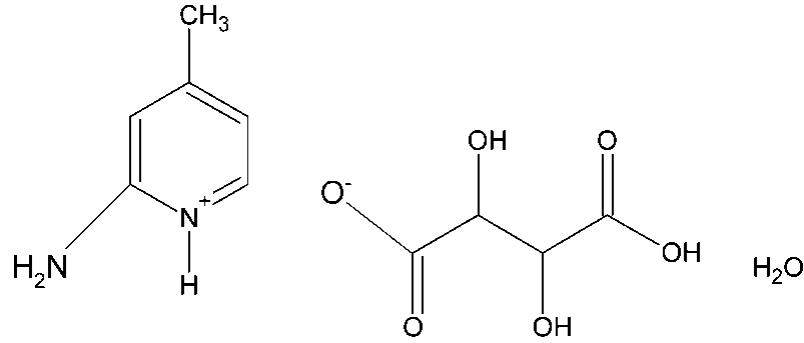


## Experimental   

### Crystal data  

C_6_H_9_N_2_^+^·C_4_H_5_O_6_^−^·H_2_O
*M* *_r_* = 276.25
Triclinic, 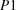

*a* = 7.176 (3) Å
*b* = 9.9359 (18) Å
*c* = 10.716 (2) Å
α = 117.528 (5)°
β = 104.792 (7)°
γ = 91.701 (7)°
*V* = 645.3 (3) Å^3^
*Z* = 2
Mo *K*α radiation
μ = 0.12 mm^−1^
*T* = 293 K
0.24 × 0.22 × 0.20 mm

### Data collection  

Bruker APEXII CCD diffractometer
Absorption correction: multi-scan (*SADABS*; Bruker, 2004 ▶) *T*_min_ = 0.971, *T*_max_ = 0.976
10211 measured reflections
4999 independent reflections
4769 reflections with *I* > 2σ(*I*)
*R*_int_ = 0.022

### Refinement  

*R*[*F* ^2^ > 2σ(*F* ^2^)] = 0.031
*wR*(*F* ^2^) = 0.088
*S* = 1.08
4999 reflections
410 parameters
3 restraints
H atoms treated by a mixture of independent and constrained refinement
Δρ_max_ = 0.27 e Å^−3^
Δρ_min_ = −0.17 e Å^−3^

### 

Data collection: *APEX2* (Bruker, 2004 ▶); cell refinement: *APEX2* and *SAINT* (Bruker, 2004 ▶); data reduction: *SAINT* and *XPREP* (Bruker, 2004 ▶); program(s) used to solve structure: *SHELXS97* (Sheldrick, 2008 ▶); program(s) used to refine structure: *SHELXL97* (Sheldrick, 2008 ▶); molecular graphics: *ORTEP-3 for Windows* (Farrugia, 2012 ▶); software used to prepare material for publication: *SHELXL97*.

## Supplementary Material

Crystal structure: contains datablock(s) I, New_Global_Publ_Block. DOI: 10.1107/S160053681401842X/su2748sup1.cif

Structure factors: contains datablock(s) I. DOI: 10.1107/S160053681401842X/su2748Isup2.hkl

Supporting information file. DOI: 10.1107/S160053681401842X/su2748Isup3.cml

**Fig. 1.**
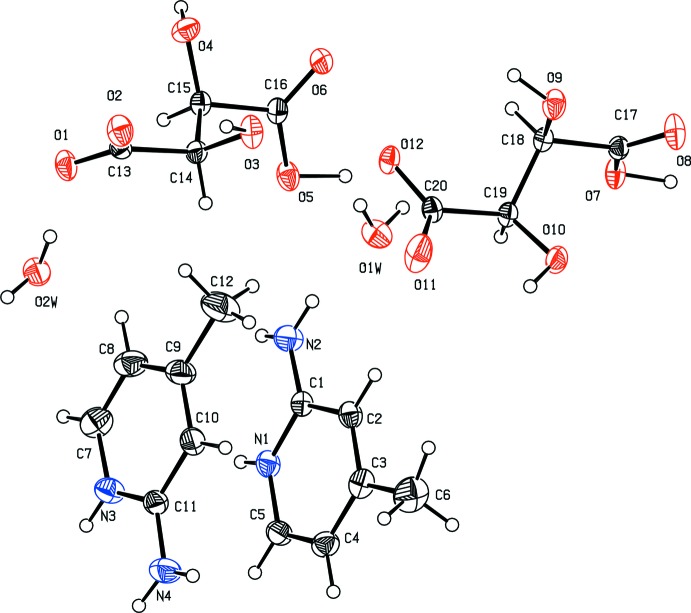
The molecular structure of the title compound, with atom labelling. Displacement ellipsoids are drawn at the 30% probability level.

**Fig. 2.**
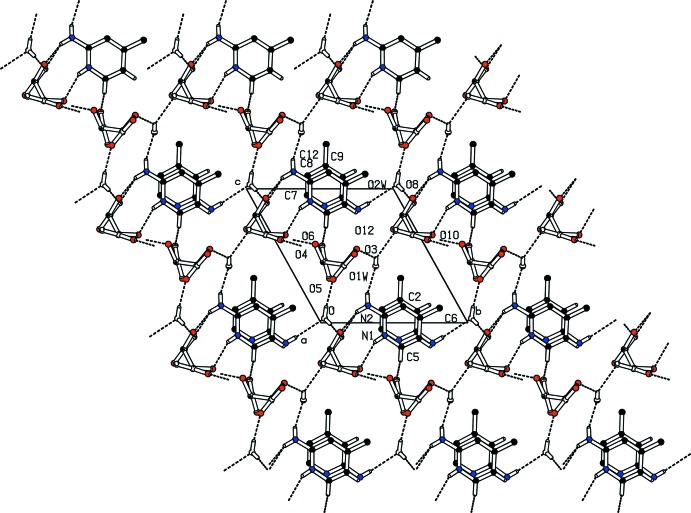
A view along the a axis of the crystal packing of the title compound. The hydrogen bonds are shown as dashed lines (see Table 1 for details).

. DOI: 10.1107/S160053681401842X/su2748fig1.tif
The mol­ecular structure of the title compound, with atom labelling. Displacement ellipsoids are drawn at the 30% probability level.

a . DOI: 10.1107/S160053681401842X/su2748fig2.tif
A view along the *a* axis of the crystal packing of the title compound. The hydrogen bonds are shown as dashed lines (see Table 1 for details).

CCDC reference: 1019274

Additional supporting information:  crystallographic information; 3D view; checkCIF report

## Figures and Tables

**Table 1:** Hydrogen-bond geometry (Å, °)

*D*—H⋯*A*	*D*—H	H⋯*A*	*D*⋯*A*	*D*—H⋯*A*
O1*W*—H1*WA*⋯O2	0.87 (3)	1.86 (3)	2.725 (3)	172 (3)
O1*W*—H1*WB*⋯O3^i^	0.78 (3)	2.39 (4)	3.012 (3)	137 (3)
O1*W*—H1*WB*⋯O12	0.78 (3)	2.39 (3)	3.006 (3)	137 (3)
O3—H3⋯O2	0.82 (4)	1.95 (4)	2.562 (2)	131 (4)
O3—H3⋯O7	0.82 (4)	2.51 (4)	3.072 (3)	127 (3)
N2—H2*NA*⋯O1*W* ^ii^	0.84 (3)	2.00 (3)	2.819 (3)	164 (3)
N3—H3*N*⋯O4	0.84 (3)	2.15 (3)	2.939 (3)	157 (3)
N3—H3*N*⋯O6	0.84 (3)	2.44 (3)	3.047 (3)	130 (3)
N2—H2*NB*⋯O8^iii^	0.83 (4)	2.41 (3)	3.032 (3)	132 (3)
N2—H2*NB*⋯O9^iii^	0.83 (4)	2.21 (4)	2.942 (3)	147 (3)
O4—H4*A*⋯O11^iv^	0.90 (4)	1.88 (4)	2.732 (3)	156 (3)
O2*W*—H2*WA*⋯O8^i^	0.82 (4)	2.00 (4)	2.819 (3)	173 (4)
O5—H5*A*⋯O1^ii^	1.16 (4)	1.41 (4)	2.534 (2)	163 (4)
N4—H4*NA*⋯O3	0.88 (3)	2.12 (3)	2.968 (3)	163 (3)
O7—H7*A*⋯O12^ii^	1.09 (4)	1.39 (4)	2.476 (2)	174 (4)
N4—H4*NB*⋯O2*W* ^ii^	0.83 (4)	2.32 (4)	3.128 (4)	164 (3)
N4—H4*NB*⋯O12^ii^	0.83 (4)	2.60 (4)	2.955 (4)	108 (3)
O9—H9⋯O2*W*	0.91 (3)	1.81 (3)	2.715 (3)	172 (3)
O10—H10*A*⋯O6^v^	0.83 (3)	2.19 (3)	2.912 (3)	145 (3)
O10—H10*A*⋯O11	0.83 (3)	2.15 (3)	2.648 (3)	119 (3)
N1—H29⋯O10^iii^	0.78 (3)	2.24 (3)	2.946 (3)	151 (3)
C5—H5⋯O6^vi^	0.93	2.46	3.158 (3)	132

Symmetry codes: (i) 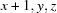; (ii) 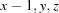; (iii) 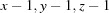; (iv) 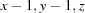; (v) 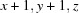; (vi) 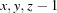.

## supplementary crystallographic information

### S1. Experimental

The title compound was prepared by mixing an equimolar ratio of 4-methylpyridin-2-amine (Aldrich) with L-(+)-tartaric acid (Spectrochem). When the tartaric acid was added to a saturated solution of 4-methylpyridin-2-amine in acetone a pale pink coloured precipitate was formed. The precipitate was collected and allowed to dry and powdered. Suitable crystals of the title salt were prepared by slow evaporation of a solution of the powder in methanol at room temperature.

### S2. Refinement

The water, NH and OH H atoms were located from difference Fourier maps and freely refined. The C-bound H atoms were positioned geometrically and treated as riding atoms: C—H = 0.93–0.98 Å *U*_iso_(H)= 1.5*U*_eq_(*C*-methyl) and = 1.2*U*_eq_(C) for other H atoms.

### Crystal data

**Table:**

C_6_H_9_N_2_^+^·C_4_H_5_O_6_^−^·H_2_O	*Z* = 2
*M**_r_* = 276.25	*F*(000) = 292
Triclinic, *P*1	*D*_x_ = 1.422 Mg m^−^^3^
Hall symbol: P 1	Mo *K*α radiation, λ = 0.71073 Å
*a* = 7.176 (3) Å	Cell parameters from 8251 reflections
*b* = 9.9359 (18) Å	θ = 2.3–32.6°
*c* = 10.716 (2) Å	µ = 0.12 mm^−^^1^
α = 117.528 (5)°	*T* = 293 K
β = 104.792 (7)°	Block, colourless
γ = 91.701 (7)°	0.24 × 0.22 × 0.20 mm
*V* = 645.3 (3) Å^3^	

### Data collection

**Table:**

Bruker APEXII CCD diffractometer	4999 independent reflections
Radiation source: fine-focus sealed tube	4769 reflections with *I* > 2σ(*I*)
Graphite monochromator	*R*_int_ = 0.022
ω scans	θ_max_ = 26.0°, θ_min_ = 2.3°
Absorption correction: multi-scan (*SADABS*; Bruker, 2004)	*h* = −8→8
*T*_min_ = 0.971, *T*_max_ = 0.976	*k* = −12→12
10211 measured reflections	*l* = −13→13

### Refinement

**Table:**

Refinement on *F*^2^	Secondary atom site location: difference Fourier map
Least-squares matrix: full	Hydrogen site location: inferred from neighbouring sites
*R*[*F*^2^ > 2σ(*F*^2^)] = 0.031	H atoms treated by a mixture of independent and constrained refinement
*wR*(*F*^2^) = 0.088	*w* = 1/[σ^2^(*F*_o_^2^) + (0.0479*P*)^2^ + 0.1123*P*] where *P* = (*F*_o_^2^ + 2*F*_c_^2^)/3
*S* = 1.08	(Δ/σ)_max_ = 0.001
4999 reflections	Δρ_max_ = 0.27 e Å^−^^3^
410 parameters	Δρ_min_ = −0.17 e Å^−^^3^
3 restraints	Extinction correction: *SHELXL*, Fc^*^=kFc[1+0.001xFc^2^λ^3^/sin(2θ)]^-1/4^
Primary atom site location: structure-invariant direct methods	Extinction coefficient: 0.055 (5)

### Special details

**Table:**

Geometry. Bond distances, angles etc. have been calculated using the rounded fractional coordinates. All su's are estimated from the variances of the (full) variance-covariance matrix. The cell esds are taken into account in the estimation of distances, angles and torsion angles
Refinement. Refinement of *F*^2^ against ALL reflections. The weighted *R*-factor *wR* and goodness of fit *S* are based on *F*^2^, conventional *R*-factors *R* are based on *F*, with *F* set to zero for negative *F*^2^. The threshold expression of *F*^2^ > σ(*F*^2^) is used only for calculating *R*-factors(gt) *etc*. and is not relevant to the choice of reflections for refinement. *R*-factors based on *F*^2^ are statistically about twice as large as those based on *F*, and *R*- factors based on ALL data will be even larger.

### Fractional atomic coordinates and isotropic or equivalent isotropic displacement parameters (Å^2^)

**Table:**

	*x*	*y*	*z*	*U*_iso_*/*U*_eq_	
O1	0.74569 (19)	0.24776 (17)	0.32479 (15)	0.0425 (4)	
O2	0.8383 (2)	0.48800 (17)	0.51261 (16)	0.0434 (4)	
O3	0.4999 (2)	0.52134 (16)	0.54862 (17)	0.0421 (5)	
O4	0.5543 (2)	0.25906 (18)	0.58628 (16)	0.0402 (4)	
O5	0.09901 (19)	0.22642 (16)	0.33245 (15)	0.0385 (4)	
O6	0.1808 (2)	0.2985 (2)	0.57363 (17)	0.0469 (5)	
O7	0.73206 (18)	0.84899 (15)	0.69807 (16)	0.0366 (4)	
O8	0.8096 (2)	1.08215 (17)	0.89449 (17)	0.0443 (4)	
O9	1.1844 (2)	1.09612 (17)	0.92901 (15)	0.0408 (4)	
O10	1.0198 (2)	1.08903 (18)	0.65507 (18)	0.0440 (5)	
O11	1.3678 (3)	1.0395 (2)	0.6194 (3)	0.0676 (8)	
O12	1.38559 (17)	0.86225 (14)	0.69019 (15)	0.0340 (4)	
C13	0.7165 (2)	0.3699 (2)	0.42355 (19)	0.0302 (5)	
C14	0.5072 (3)	0.3760 (2)	0.4327 (2)	0.0299 (5)	
C15	0.4308 (2)	0.2468 (2)	0.4546 (2)	0.0295 (5)	
C16	0.2212 (2)	0.25924 (19)	0.4604 (2)	0.0298 (5)	
C17	0.8533 (2)	0.9703 (2)	0.80181 (19)	0.0286 (5)	
C18	1.0658 (2)	0.9648 (2)	0.80165 (19)	0.0275 (5)	
C19	1.0873 (2)	0.95937 (19)	0.66195 (19)	0.0290 (5)	
C20	1.2990 (2)	0.9559 (2)	0.65682 (19)	0.0300 (5)	
N1	0.0123 (3)	0.3863 (2)	−0.09469 (18)	0.0368 (5)	
N2	0.0764 (3)	0.3843 (2)	0.1268 (2)	0.0444 (6)	
C1	0.0410 (3)	0.4600 (2)	0.0521 (2)	0.0316 (5)	
C2	0.0344 (3)	0.6200 (2)	0.1196 (2)	0.0355 (5)	
C3	0.0001 (3)	0.6933 (2)	0.0375 (2)	0.0391 (6)	
C4	−0.0325 (3)	0.6082 (3)	−0.1162 (2)	0.0417 (7)	
C5	−0.0249 (3)	0.4564 (2)	−0.1787 (2)	0.0397 (6)	
C6	−0.0012 (5)	0.8623 (3)	0.1096 (3)	0.0639 (9)	
N3	0.4969 (3)	0.4655 (2)	0.8719 (2)	0.0450 (6)	
N4	0.4515 (3)	0.6816 (3)	0.8476 (3)	0.0535 (8)	
O1W	1.1106 (3)	0.58916 (19)	0.42444 (17)	0.0448 (5)	
C7	0.5351 (4)	0.3913 (3)	0.9520 (3)	0.0528 (8)	
C8	0.5627 (4)	0.4668 (3)	1.0974 (3)	0.0529 (8)	
C9	0.5523 (3)	0.6239 (3)	1.1678 (2)	0.0499 (8)	
C10	0.5161 (3)	0.6983 (3)	1.0861 (2)	0.0426 (6)	
C11	0.4877 (3)	0.6164 (2)	0.9329 (2)	0.0367 (6)	
C12	0.5813 (6)	0.7111 (5)	1.3315 (3)	0.0825 (13)	
O2W	1.5440 (2)	1.04131 (19)	1.03282 (18)	0.0444 (5)	
H3	0.613 (5)	0.565 (4)	0.575 (4)	0.067 (9)*	
H4A	0.513 (5)	0.170 (4)	0.582 (3)	0.062 (8)*	
H5A	−0.057 (6)	0.230 (4)	0.344 (4)	0.098 (12)*	
H14	0.42260	0.36470	0.33960	0.0360*	
H15	0.43150	0.14750	0.37120	0.0350*	
H7A	0.582 (5)	0.862 (4)	0.698 (4)	0.082 (10)*	
H9	1.300 (4)	1.068 (3)	0.959 (3)	0.055 (7)*	
H10A	1.091 (4)	1.116 (3)	0.618 (3)	0.053 (7)*	
H18	1.10350	0.87210	0.80480	0.0330*	
H19	1.00390	0.86590	0.57650	0.0350*	
H2	0.05370	0.67540	0.22070	0.0430*	
H2NA	0.085 (3)	0.429 (3)	0.217 (3)	0.029 (5)*	
H4	−0.05890	0.65630	−0.17380	0.0500*	
H2NB	0.071 (5)	0.290 (4)	0.081 (3)	0.058 (8)*	
H5	−0.04540	0.39950	−0.27990	0.0480*	
H6A	0.09330	0.91180	0.08800	0.0960*	
H6B	0.03140	0.90320	0.21460	0.0960*	
H6C	−0.12900	0.88070	0.07290	0.0960*	
H29	0.015 (4)	0.299 (3)	−0.138 (3)	0.047 (7)*	
H3N	0.481 (4)	0.412 (3)	0.781 (3)	0.048 (7)*	
H7	0.54230	0.28690	0.90550	0.0630*	
H4NA	0.441 (4)	0.624 (3)	0.754 (3)	0.045 (7)*	
H8	0.58890	0.41520	1.15170	0.0630*	
H4NB	0.452 (5)	0.776 (4)	0.890 (4)	0.068 (10)*	
H10	0.51030	0.80290	1.13180	0.0510*	
H12A	0.70470	0.69940	1.38340	0.1240*	
H12B	0.47770	0.67110	1.35330	0.1240*	
H12C	0.57980	0.81830	1.36210	0.1240*	
H1WA	1.033 (4)	0.554 (3)	0.457 (3)	0.052 (7)*	
H1WB	1.211 (5)	0.620 (3)	0.487 (3)	0.055 (8)*	
H2WA	1.617 (5)	1.059 (4)	0.992 (3)	0.062 (9)*	
H2WB	1.591 (4)	1.099 (3)	1.125 (4)	0.051 (7)*	

### Atomic displacement parameters (Å^2^)

**Table:**

	*U*^11^	*U*^22^	*U*^33^	*U*^12^	*U*^13^	*U*^23^
O1	0.0253 (6)	0.0529 (9)	0.0359 (7)	0.0069 (6)	0.0113 (5)	0.0098 (7)
O2	0.0303 (7)	0.0451 (8)	0.0468 (8)	−0.0004 (6)	0.0125 (6)	0.0160 (7)
O3	0.0397 (8)	0.0312 (7)	0.0588 (9)	0.0085 (6)	0.0267 (7)	0.0185 (7)
O4	0.0343 (7)	0.0467 (8)	0.0487 (8)	0.0063 (6)	0.0085 (6)	0.0326 (7)
O5	0.0257 (6)	0.0419 (7)	0.0344 (7)	0.0021 (5)	0.0100 (5)	0.0076 (6)
O6	0.0457 (8)	0.0691 (10)	0.0530 (9)	0.0287 (8)	0.0309 (7)	0.0421 (8)
O7	0.0240 (6)	0.0291 (6)	0.0471 (8)	0.0044 (5)	0.0135 (5)	0.0095 (6)
O8	0.0300 (7)	0.0367 (7)	0.0509 (8)	0.0049 (6)	0.0193 (6)	0.0056 (6)
O9	0.0262 (6)	0.0410 (7)	0.0380 (7)	0.0055 (6)	0.0055 (5)	0.0076 (6)
O10	0.0455 (8)	0.0532 (9)	0.0648 (9)	0.0284 (7)	0.0330 (8)	0.0444 (8)
O11	0.0513 (9)	0.0784 (12)	0.1307 (18)	0.0294 (9)	0.0552 (11)	0.0819 (13)
O12	0.0245 (6)	0.0340 (7)	0.0484 (7)	0.0084 (5)	0.0153 (5)	0.0215 (6)
C13	0.0263 (8)	0.0413 (10)	0.0292 (8)	0.0072 (8)	0.0106 (7)	0.0209 (8)
C14	0.0258 (8)	0.0355 (9)	0.0334 (9)	0.0074 (7)	0.0111 (7)	0.0196 (7)
C15	0.0262 (8)	0.0291 (8)	0.0336 (9)	0.0057 (7)	0.0115 (7)	0.0142 (7)
C16	0.0286 (9)	0.0254 (8)	0.0402 (9)	0.0063 (7)	0.0156 (7)	0.0170 (7)
C17	0.0255 (8)	0.0306 (9)	0.0342 (9)	0.0070 (7)	0.0123 (7)	0.0176 (7)
C18	0.0233 (8)	0.0249 (8)	0.0357 (9)	0.0063 (6)	0.0106 (7)	0.0150 (7)
C19	0.0242 (8)	0.0291 (9)	0.0362 (9)	0.0073 (7)	0.0117 (7)	0.0164 (8)
C20	0.0286 (8)	0.0274 (8)	0.0360 (9)	0.0053 (7)	0.0153 (7)	0.0141 (7)
N1	0.0436 (9)	0.0281 (8)	0.0353 (8)	0.0098 (7)	0.0149 (7)	0.0109 (7)
N2	0.0646 (12)	0.0312 (9)	0.0364 (10)	0.0133 (8)	0.0164 (8)	0.0149 (8)
C1	0.0290 (8)	0.0311 (9)	0.0334 (9)	0.0063 (7)	0.0106 (7)	0.0140 (8)
C2	0.0381 (10)	0.0292 (9)	0.0329 (9)	0.0061 (7)	0.0124 (8)	0.0093 (8)
C3	0.0349 (10)	0.0307 (10)	0.0507 (11)	0.0032 (8)	0.0128 (9)	0.0193 (9)
C4	0.0407 (11)	0.0459 (12)	0.0473 (11)	0.0082 (9)	0.0135 (9)	0.0295 (10)
C5	0.0385 (10)	0.0480 (12)	0.0335 (9)	0.0073 (8)	0.0123 (8)	0.0198 (9)
C6	0.0813 (19)	0.0347 (12)	0.0725 (17)	0.0119 (12)	0.0208 (15)	0.0248 (12)
N3	0.0541 (11)	0.0444 (10)	0.0342 (9)	0.0062 (8)	0.0122 (8)	0.0179 (8)
N4	0.0698 (14)	0.0596 (13)	0.0480 (12)	0.0180 (11)	0.0234 (10)	0.0365 (11)
O1W	0.0442 (8)	0.0447 (8)	0.0366 (8)	−0.0045 (7)	0.0102 (7)	0.0143 (7)
C7	0.0550 (13)	0.0454 (12)	0.0626 (15)	0.0048 (10)	0.0142 (11)	0.0320 (12)
C8	0.0500 (13)	0.0651 (15)	0.0577 (14)	0.0048 (11)	0.0122 (11)	0.0435 (13)
C9	0.0369 (11)	0.0773 (17)	0.0395 (11)	0.0048 (11)	0.0085 (9)	0.0334 (12)
C10	0.0403 (11)	0.0472 (12)	0.0404 (10)	0.0109 (9)	0.0156 (9)	0.0194 (9)
C11	0.0319 (9)	0.0461 (11)	0.0376 (10)	0.0063 (8)	0.0126 (7)	0.0238 (9)
C12	0.092 (2)	0.108 (3)	0.0447 (14)	0.019 (2)	0.0205 (15)	0.0349 (16)
O2W	0.0345 (7)	0.0524 (9)	0.0328 (8)	0.0026 (6)	0.0055 (6)	0.0125 (7)

### Geometric parameters (Å, º)

**Table:**

O1—C13	1.256 (2)	C2—C3	1.363 (3)
O2—C13	1.237 (3)	N2—H2NA	0.84 (3)
O3—C14	1.418 (3)	N2—H2NB	0.83 (4)
O4—C15	1.410 (2)	C3—C4	1.410 (3)
O5—C16	1.308 (2)	C3—C6	1.491 (4)
O6—C16	1.207 (2)	C4—C5	1.348 (4)
O3—H3	0.82 (4)	C2—H2	0.9300
O4—H4A	0.90 (4)	N3—C11	1.342 (3)
O5—H5A	1.16 (4)	N3—C7	1.354 (4)
O7—C17	1.285 (2)	N4—C11	1.326 (4)
O8—C17	1.216 (3)	C4—H4	0.9300
O9—C18	1.409 (2)	C5—H5	0.9300
O10—C19	1.417 (3)	C6—H6A	0.9600
O11—C20	1.216 (3)	C6—H6B	0.9600
O12—C20	1.268 (3)	C6—H6C	0.9600
C13—C14	1.531 (3)	N3—H3N	0.84 (3)
C14—C15	1.519 (3)	N4—H4NB	0.83 (4)
C15—C16	1.527 (2)	N4—H4NA	0.88 (3)
O7—H7A	1.09 (4)	C7—C8	1.335 (4)
O9—H9	0.91 (3)	C8—C9	1.399 (4)
O10—H10A	0.83 (3)	C9—C10	1.369 (4)
C14—H14	0.9800	C9—C12	1.506 (3)
C15—H15	0.9800	C10—C11	1.408 (3)
C17—C18	1.528 (2)	O1W—H1WB	0.78 (3)
C18—C19	1.520 (3)	O1W—H1WA	0.87 (3)
C19—C20	1.535 (2)	C7—H7	0.9300
N1—C5	1.355 (3)	C8—H8	0.9300
N1—C1	1.347 (3)	C10—H10	0.9300
N2—C1	1.317 (3)	C12—H12C	0.9600
C18—H18	0.9800	C12—H12A	0.9600
C19—H19	0.9800	C12—H12B	0.9600
C1—C2	1.418 (3)	O2W—H2WA	0.82 (4)
N1—H29	0.78 (3)	O2W—H2WB	0.84 (4)
			
C14—O3—H3	100 (3)	C1—N2—H2NB	119 (2)
C15—O4—H4A	103.0 (19)	C1—C2—C3	120.58 (17)
C16—O5—H5A	108.0 (18)	H2NA—N2—H2NB	121 (3)
O1—C13—C14	116.99 (16)	C2—C3—C4	119.4 (2)
O1—C13—O2	126.88 (16)	C4—C3—C6	120.2 (2)
O2—C13—C14	116.10 (17)	C2—C3—C6	120.37 (19)
O3—C14—C13	109.65 (16)	C3—C4—C5	119.3 (2)
O3—C14—C15	110.52 (16)	N1—C5—C4	120.27 (18)
C13—C14—C15	111.91 (17)	C1—C2—H2	120.00
C14—C15—C16	108.80 (17)	C3—C2—H2	120.00
O4—C15—C14	108.89 (16)	C7—N3—C11	122.6 (2)
O4—C15—C16	111.36 (15)	C5—C4—H4	120.00
O5—C16—O6	126.04 (16)	C3—C4—H4	120.00
O5—C16—C15	112.27 (15)	N1—C5—H5	120.00
O6—C16—C15	121.67 (16)	C4—C5—H5	120.00
C17—O7—H7A	111 (2)	C3—C6—H6B	109.00
C18—O9—H9	107.2 (19)	C3—C6—H6C	109.00
C19—O10—H10A	106 (2)	C3—C6—H6A	109.00
O3—C14—H14	108.00	H6A—C6—H6C	110.00
C13—C14—H14	108.00	H6B—C6—H6C	109.00
C15—C14—H14	108.00	H6A—C6—H6B	109.00
C16—C15—H15	109.00	C11—N3—H3N	121 (2)
O4—C15—H15	109.00	C7—N3—H3N	116 (2)
C14—C15—H15	109.00	C11—N4—H4NA	118 (2)
O7—C17—C18	114.13 (16)	H4NA—N4—H4NB	126 (3)
O7—C17—O8	125.05 (16)	C11—N4—H4NB	116 (3)
O8—C17—C18	120.83 (17)	N3—C7—C8	120.5 (3)
O9—C18—C17	108.17 (14)	C7—C8—C9	119.9 (3)
O9—C18—C19	111.32 (16)	C8—C9—C12	120.8 (3)
C17—C18—C19	109.84 (14)	C10—C9—C12	120.1 (3)
C18—C19—C20	112.12 (14)	C8—C9—C10	119.2 (2)
O10—C19—C18	108.04 (15)	C9—C10—C11	120.0 (2)
O10—C19—C20	111.07 (16)	N3—C11—C10	117.8 (2)
O11—C20—O12	125.81 (18)	N4—C11—C10	122.9 (2)
O11—C20—C19	118.3 (2)	N3—C11—N4	119.2 (2)
O12—C20—C19	115.93 (17)	H1WA—O1W—H1WB	105 (3)
C1—N1—C5	123.5 (2)	N3—C7—H7	120.00
C17—C18—H18	109.00	C8—C7—H7	120.00
C19—C18—H18	109.00	C9—C8—H8	120.00
O9—C18—H18	109.00	C7—C8—H8	120.00
C18—C19—H19	109.00	C9—C10—H10	120.00
C20—C19—H19	108.00	C11—C10—H10	120.00
O10—C19—H19	109.00	C9—C12—H12B	109.00
N1—C1—N2	120.2 (2)	C9—C12—H12C	109.00
N1—C1—C2	116.89 (19)	C9—C12—H12A	109.00
N2—C1—C2	122.93 (18)	H12A—C12—H12C	110.00
C5—N1—H29	115 (2)	H12B—C12—H12C	109.00
C1—N1—H29	122 (2)	H12A—C12—H12B	109.00
C1—N2—H2NA	120 (2)	H2WA—O2W—H2WB	109 (3)
			
O1—C13—C14—O3	−179.57 (18)	C18—C19—C20—O11	−135.1 (2)
O1—C13—C14—C15	57.4 (2)	C18—C19—C20—O12	46.2 (2)
O2—C13—C14—O3	−1.2 (3)	C5—N1—C1—N2	180.0 (2)
O2—C13—C14—C15	−124.3 (2)	C5—N1—C1—C2	−1.1 (3)
O3—C14—C15—O4	−63.3 (2)	C1—N1—C5—C4	0.9 (4)
O3—C14—C15—C16	58.3 (2)	N2—C1—C2—C3	179.0 (2)
C13—C14—C15—O4	59.26 (19)	N1—C1—C2—C3	0.1 (3)
C13—C14—C15—C16	−179.20 (14)	C1—C2—C3—C4	1.2 (3)
O4—C15—C16—O5	−170.89 (18)	C1—C2—C3—C6	−178.3 (2)
O4—C15—C16—O6	10.5 (3)	C6—C3—C4—C5	178.0 (2)
C14—C15—C16—O5	69.1 (2)	C2—C3—C4—C5	−1.4 (3)
C14—C15—C16—O6	−109.5 (2)	C3—C4—C5—N1	0.5 (3)
O7—C17—C18—O9	−173.74 (17)	C11—N3—C7—C8	1.0 (4)
O7—C17—C18—C19	64.6 (2)	C7—N3—C11—N4	179.3 (2)
O8—C17—C18—O9	6.1 (3)	C7—N3—C11—C10	−1.1 (4)
O8—C17—C18—C19	−115.6 (2)	N3—C7—C8—C9	−0.1 (4)
O9—C18—C19—O10	−63.25 (18)	C7—C8—C9—C12	179.5 (3)
O9—C18—C19—C20	59.5 (2)	C7—C8—C9—C10	−0.8 (4)
C17—C18—C19—O10	56.5 (2)	C8—C9—C10—C11	0.7 (3)
C17—C18—C19—C20	179.23 (17)	C12—C9—C10—C11	−179.6 (3)
O10—C19—C20—O11	−14.1 (3)	C9—C10—C11—N4	179.8 (2)
O10—C19—C20—O12	167.13 (16)	C9—C10—C11—N3	0.2 (3)

### Hydrogen-bond geometry (Å, º)

**Table:**

*D*—H···*A*	*D*—H	H···*A*	*D*···*A*	*D*—H···*A*
O1*W*—H1*WA*···O2	0.87 (3)	1.86 (3)	2.725 (3)	172 (3)
O1*W*—H1*WB*···O3^i^	0.78 (3)	2.39 (4)	3.012 (3)	137 (3)
O1*W*—H1*WB*···O12	0.78 (3)	2.39 (3)	3.006 (3)	137 (3)
O3—H3···O2	0.82 (4)	1.95 (4)	2.562 (2)	131 (4)
O3—H3···O7	0.82 (4)	2.51 (4)	3.072 (3)	127 (3)
N2—H2*NA*···O1*W*^ii^	0.84 (3)	2.00 (3)	2.819 (3)	164 (3)
N3—H3*N*···O4	0.84 (3)	2.15 (3)	2.939 (3)	157 (3)
N3—H3*N*···O6	0.84 (3)	2.44 (3)	3.047 (3)	130 (3)
N2—H2*NB*···O8^iii^	0.83 (4)	2.41 (3)	3.032 (3)	132 (3)
N2—H2*NB*···O9^iii^	0.83 (4)	2.21 (4)	2.942 (3)	147 (3)
O4—H4*A*···O11^iv^	0.90 (4)	1.88 (4)	2.732 (3)	156 (3)
O2*W*—H2*WA*···O8^i^	0.82 (4)	2.00 (4)	2.819 (3)	173 (4)
O5—H5*A*···O1^ii^	1.16 (4)	1.41 (4)	2.534 (2)	163 (4)
N4—H4*NA*···O3	0.88 (3)	2.12 (3)	2.968 (3)	163 (3)
O7—H7*A*···O12^ii^	1.09 (4)	1.39 (4)	2.476 (2)	174 (4)
N4—H4*NB*···O2*W*^ii^	0.83 (4)	2.32 (4)	3.128 (4)	164 (3)
N4—H4*NB*···O12^ii^	0.83 (4)	2.60 (4)	2.955 (4)	108 (3)
O9—H9···O2*W*	0.91 (3)	1.81 (3)	2.715 (3)	172 (3)
O10—H10*A*···O6^v^	0.83 (3)	2.19 (3)	2.912 (3)	145 (3)
O10—H10*A*···O11	0.83 (3)	2.15 (3)	2.648 (3)	119 (3)
N1—H29···O10^iii^	0.78 (3)	2.24 (3)	2.946 (3)	151 (3)
C5—H5···O6^vi^	0.93	2.46	3.158 (3)	132

Symmetry codes: (i) *x*+1, *y*, *z*; (ii) *x*−1, *y*, *z*; (iii) *x*−1, *y*−1, *z*−1; (iv) *x*−1, *y*−1, *z*; (v) *x*+1, *y*+1, *z*; (vi) *x*, *y*, *z*−1.
