# Supplementary figures and images for: Crystal structure of 2-amino-4-methyl­pyridin-1-ium (2R,3R)-3-carb­oxy-2,3-di­hydroxy­propano­ate monohydrate
Source: Acta Crystallogr Sect E Struct Rep Online. 2014 Aug 23;70(Pt 9):o1036–7. doi: 10.1107/S160053681401842X (PMC4186070; doi:10.1107/S160053681401842X)

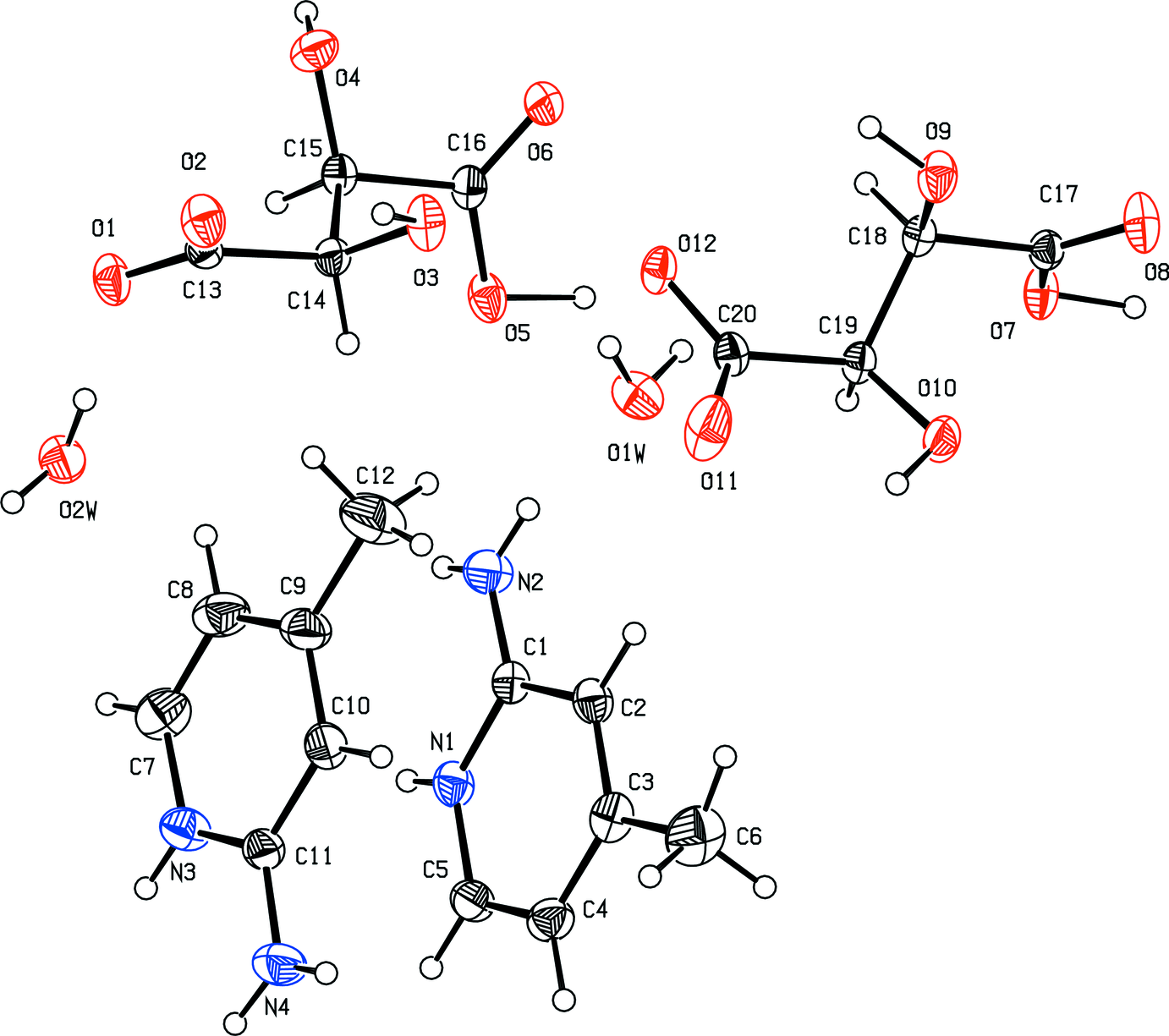

Supplement: Supplementary file 4 [file e-70-o1036-fig1.tif]

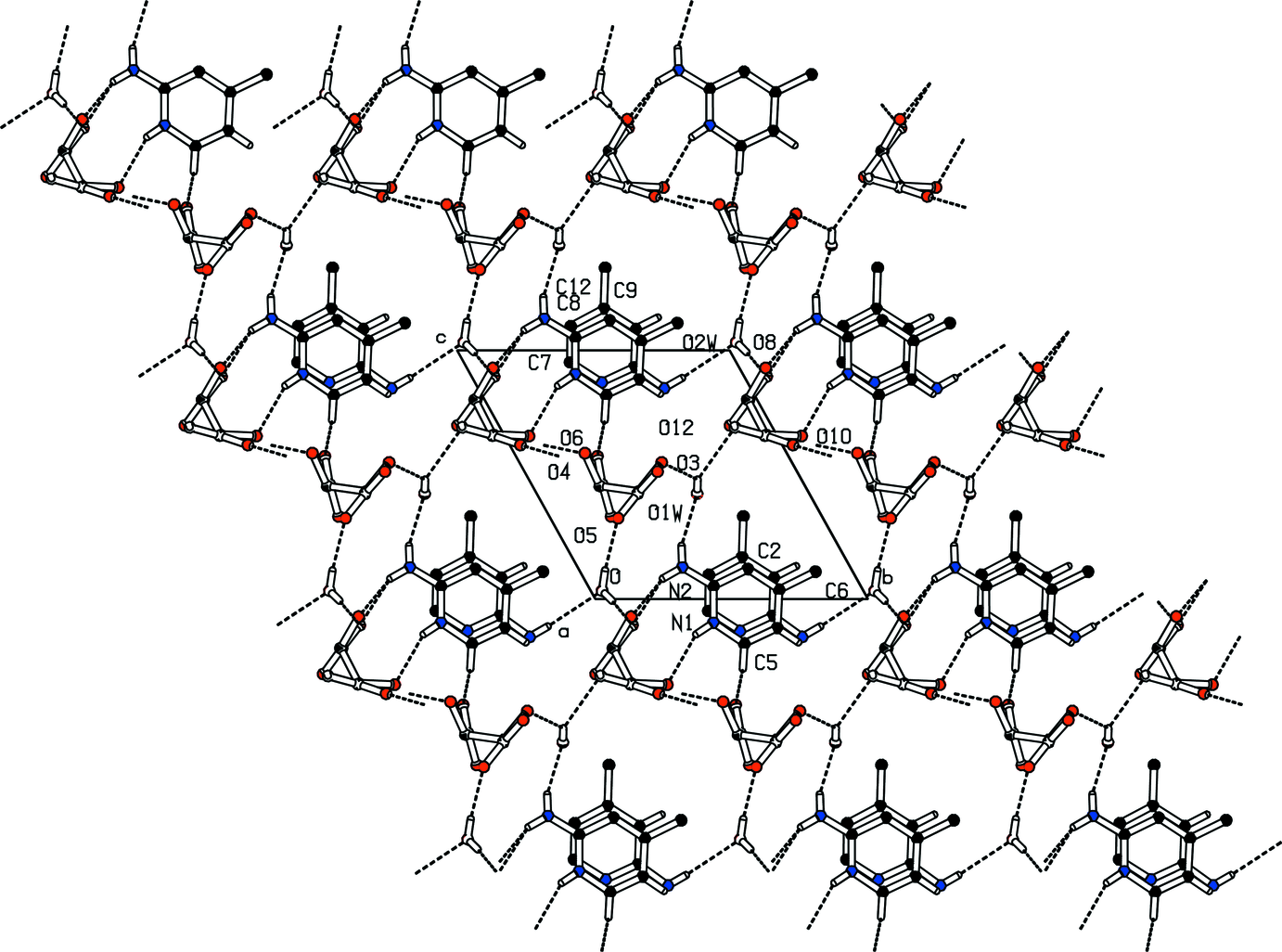

Supplement: Supplementary file 5 [file e-70-o1036-fig2.tif]
